# Multiplane differential phase contrast imaging using asymmetric illumination in volume holographic microscopy

**DOI:** 10.1117/1.JBO.25.12.123704

**Published:** 2020-11-27

**Authors:** Yu-Hsin Chia, Sunil Vyas, Jui-Chang Tsai, Yi-You Huang, J. Andrew Yeh, Yuan Luo

**Affiliations:** a National Taiwan University, Institute of Medical Device and Imaging, Taipei, Taiwan; b National Taiwan University, Department of Biomedical Engineering, Taipei, Taiwan; c National Taiwan University Hospital, Department of Biomedical Engineering, Taipei, Taiwan; d National Tsing Hua University, Department of Power Mechanical Engineering, Hsinchu, Taiwan; e National Taiwan University, Molecular Imaging Center, Taipei, Taiwan; f National Taiwan University, YongLin Institute of Health, Taipei, Taiwan

**Keywords:** diffraction gratings, volume gratings, holography, imaging systems, microscopy, illumination design

## Abstract

**Significance:** Differential phase contrast (DPC) is a well-known imaging technique for phase imaging. However, simultaneously acquiring multidepth DPC images is a non-trivial task. We propose simultaneous multiplane DPC imaging using volume holographic microscopy (VHM).

**Aim:** To design and implement a new configuration of DPC-VHM for multiplane imaging.

**Approach:** The angularly multiplexed volume holographic gratings (AMVHGs) and the wavelength-coded volume holographic gratings (WC-VHGs) are used for this purpose. To obtain asymmetric illumination for DPC images, a dynamic illumination system is designed by modifying the regular Köhler illumination using a thin film transistor panel (TFT-panel).

**Results:** Multidepth DPC images of standard resolution chart and biosamples were used to compare imaging performance with the corresponding bright-field images. An average contrast enhancement of around three times is observed for target resolution chart by DPC-VHM. Imaging performance of our system is studied by modulation transfer function analysis, which suggests that DPC-VHM not only suppresses the DC component but also enhances high-frequency information.

**Conclusions:** Proposed DPC-VHM can acquire multidepth-resolved DPC images without axial scanning. The illumination part of the system is adjustable so that the system can be adapted to bright-field mode, phase contrast mode, and DPC mode by controlling the pattern on the TFT-panel.

## 1 Introduction

Recently, phase imaging techniques have seen renewed interest mainly due to advances in computational imaging methods.^1^ Transparent specimens have weak phase features, which are difficult to capture by most conventional microscopes.^2^ A variety of imaging techniques have been developed to observe phase objects; among them, Schlieren method and central dark ground method are prominent.^3^ Schlieren method requires a knife-edge object at the Fourier plane for a phase image formation, which is difficult to align at the correct Fourier plane, and reproducibility of results is an important issue.^4^ In the central dark ground method, an annular aperture is used to block out the DC term of light, which results in significant loss of intensity of the image.^5^ To overcome these issues, the differential phase contrast (DPC) technique has been proposed. Schlieren method and central dark ground method are limited by the same drawback: the observed intensity variations are not linearly related to the phase shift generated by the object. Previous studies of phase imaging show that the DPC performs better than the above methods. The above method cannot provide the multidepth phase contrast images. On the other hand, phase contrast microscopy is a well-established technique and it has been regularly used to obtain the image contrast to find inner structural details of transparent objects without any fluorescence labeling.^1^ DPC is one of the contrast enhancement techniques and offers many advantages over its counterparts. Various optical systems have been designed to obtain DPC images by conventional wide-field microscopes.^6^^,^^7^ The DPC technique can provide the volumetric phase information of the object. After using the 4 half-circle pattern asymmetric illuminations to capture the multidepth images of the weak object, the technique utilizes the global fast Fourier transformation-based method to provide the 3D phase images. However, the 3D DPC techniques cannot take the multifocus planes images without the axial scanning. In addition, DPC methods have also been used for quantitative phase imaging. Various kinds of illumination schemes using pupil function engineering and phase retrieval methods were used to measure quantitative phase for a variety of biosamples.^8^^–^^10^ However, the 3D DPC techniques cannot take the multifocus planes images without the axial scanning, it still requires the mechanical or optical scanning mechanism. Acquisition of accurate multidepth DPC images with wide-field microscope is a non-trivial task. Nevertheless, most of the multidimensional imaging systems require mechanical or electro-optic axial scanning mechanism to construct multidepth images.

In the past decades, volume holographic systems have been developed for multidimensional imaging. The main idea in various volume holographic imaging systems is to reduce the requirements of axial scanning and to acquire multidepth images directly.^11^ In general, a volume holographic microscope consists of a 4-f imaging system with multiplexed volume holographic gratings at the Fourier plane to simultaneously observe multidepth images of a specimen. In addition, it has been demonstrated that volume holographic microscopy (VHM) works well with the coherent as well as incoherent light sources.^11^^–^^14^ Although phase contrast VHM techniques have been proposed in past to enhance phase information of the objects, there is still large scope for their improvement in functionality. There are mainly two VHM techniques for getting phase contrast in which either a knife-edge component is used in the imaging or in the recording system.^12^^,^^13^ Former method uses a knife-edge filter at the conjugate plane of the volume hologram pupil to block half spatial frequency components at the Fourier plane to obtain phase contrast image. In the latter method, a knife-edge filter needs to be placed in front of the volume hologram during the recording process. During imaging, these gratings enhance the high spatial frequency information of Fourier spectrum to generate a phase contrast images.

In this paper, we propose and experimentally demonstrate the DPC imaging technique in VHM using asymmetric illumination. We present two new configurations for DPC-VHM systems. Unlike previously mentioned volume holographic microscope systems that use uniform illumination of object and hence cannot provide the DPC images,^12^^,^^13^ our system utilizes specially designed asymmetrical illumination patterns to get the DPC images.^15^ In the first configuration, a pair-wise alternate black and white half-circle amplitude mask directly helps in performing DPC operation, whereas in the second configuration DPC is achieved by two color (red–blue) amplitude mask as an asymmetric illumination. In both the configurations, the thin film transistor panel (TFT-panel) can be dynamically controlled to generate different amplitude patterns for asymmetric illumination. In the limiting condition, by just displaying complete white pattern the present system can also work as a bright-field volume holographic microscope.^11^

## 2 Methods

### 2.1 Differential Phase Contrast

In our imaging systems, DPC images are obtained by the method proposed by Dekkers and de Lang.^16^^,^^17^ The complex transmittance of the sample can be written as

t(x)=a(x)exp jϕ(x). (1)

If the pair of the asymmetric illumination images are added together, then the amplitude information can be obtained as

$$I_{+}(x)=I_{1,1}+I_{1,2}=a^{2}(x),$$ (2)

while if they are subtracted, they give the differential of the phase modulated by $\alpha^{2}(x)$

$$I_{-}(x)=I_{1,1}-I_{1,2}=a^{2}(x)\frac{d\phi }{dx}.$$ (3)

By Eqs. (2) and (3), differential of the phase can be obtained as

$$\frac{d\phi }{dx}=\frac{I_{-}(x)}{I_{+}(x)}.$$ (4)

The differential of phase is sensitive to the phase variations, which results in contrast enhancement of high spatial frequencies as well as suppression of low frequencies.

Here, we are following the DPC method based on the asymmetric illumination.^16^^,^^17^ The black and white half-circle pattern for the asymmetric illumination at the Fourier plane can be expressed as

$$H_{1,1}=\frac{1}{2}[1+\mathrm{sgn}(f_{x})],$$ (5)

$$H_{1,2}=\frac{1}{2}[1+\mathrm{sgn}(f_{x})],$$ (6)

where $H_{1}$ and $H_{2}$ are the transfer function of the right- and left-hand side circles, respectively, $f_{x}$ is the coordinate on the Fourier plane along the x direction, and sgn is the sign function.^12^ The complex transmittance of the weak phase sample in one direction (x) can be expressed as

t(x)=exp jϕ(x)=1+jϕ(x), (7)

where ϕ(x) is the phase of the sample in the x direction. The images of weak phase sample taken with asymmetric illumination can be obtained as

$$I_{1,1}={\left|t(x)*FT[H_{1}]\right|}^{2}={\left|[1+j\phi (x)]*FT[\frac{1+\mathrm{sgn}(f_{x})}{2}]\right|}^{2}\;=1+2(\phi (x)*\frac{1}{\pi x})+\phi {\left(x\right)}^{2}+{\left(\phi (x)*\frac{1}{\pi x}\right)}^{2},$$ (8)

$$I_{1,2}={\left|t(x)*FT[H_{2}]\right|}^{2}={\left|[1+j\phi (x)]*\frac{1}{2}[\delta (x)-\frac{1}{j\pi x}]\right|}^{2}\;=1-2(\phi (x)*\frac{1}{\pi x})+\phi {\left(x\right)}^{2}-{\left(\phi (x)*\frac{1}{\pi x}\right)}^{2},$$ (9)

where $I_{1,1}$ and $I_{1,2}$ are the observed images captured by the right and left half-circles illumination, respectively, and FT means the Fourier transform, and * represents the convolution operation. If the pair-wise asymmetric illumination images ($I_{1,1}$ and $I_{1,2}$) are added together, then the final image can be obtained as

$$I_{+}=I_{1,1}+I_{1,2}=2(1+\phi (x{)}^{2}),$$ (10)

where the ϕ(x) is small and can be ignored for simplicity, thus the $I_{+}$ can be regarded as a constant value. While if pair-wise asymmetric illumination images are subtracted, then the final image can be obtained as

$$I_{-}=I_{1,1}-I_{1,2}=4(\phi (x)*\frac{1}{\pi x})+2(\phi (x)*\frac{1}{\pi x}{)}^{2}.$$ (11)

Next if we make above two equations to divide with each other, we can obtain

$$\frac{I_{-}}{I_{+}}=\frac{2(\phi (x)*\frac{1}{\pi x})+{\left(\phi (x)*\frac{1}{\pi x}\right)}^{2}}{\left(1+\phi {\left(x\right)}^{2}\right)}\alpha (\phi (x)*\frac{1}{\pi x}),$$ (12)

where $\left(\phi (x)*\frac{1}{\pi x}\right)$ is the Hilbert transform of the ϕ(x). According to the property of Hilbert transform, our DPC-VHM can suppress the low-frequencies DC term and enhance the high-frequencies component with huge phase variation.

### 2.2 AMVHGs Based DPC-VHM

**Fig. 1:**
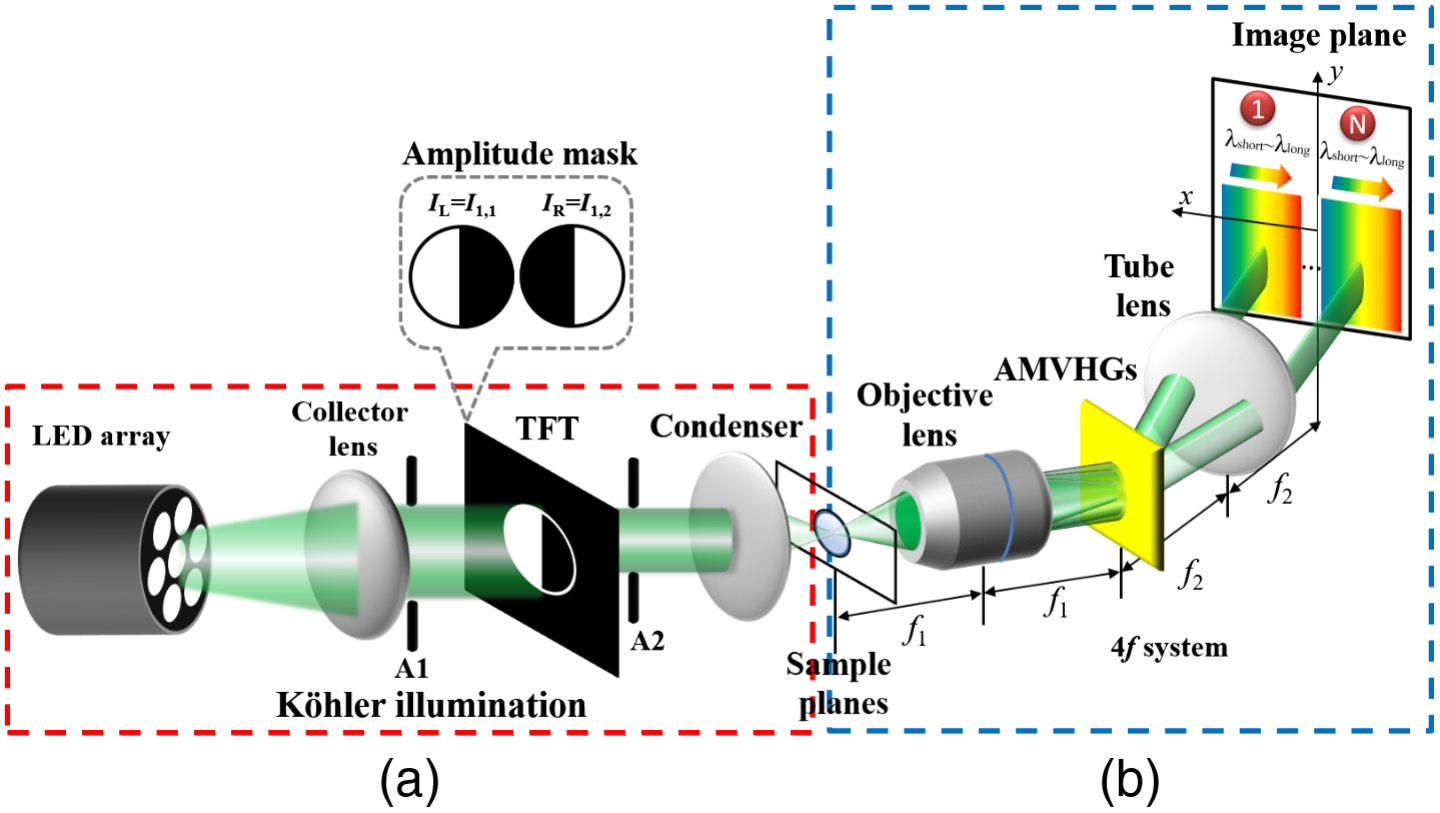
Schematic diagram of the proposed DPC-VHM with Köhler illumination. (a) Asymmetric illumination within the highlighted red dashed box and (b) AMVHGs imaging within the highlighted blue dashed box (TFT, thin film transistor panel; AMVHGs, angularly multiplexed volume holographic gratings).

The first configuration of DPC-VHM has two main parts, asymmetric illumination system and an angularly multiplexed volume holographic gratings (AMVHGs) imaging system as shown in Fig. 1. Asymmetric amplitude patterns are obtained by a special Köhler illumination setup, which consists of a broadband green LED light source (LIU525B, THORLAB, λ=525  nm, Δλ∼80  nm), a collector lens (f=15  cm), a condenser lens (f=2.54  cm), and a TFT-panel (ILI9486, 480×320  pixels, pixel size=15  μm). The panel is located at the front focal plane of the condenser lens. The function of the TFT-panel is to dynamically generate asymmetrical illumination pattern. Two black and white half-circle aperture patterns ($I_{L}$ and $I_{R}$) are sequentially projected to obtain two corresponding images for DPC operation. The imaging part of DPC-VHM consist of an objective (ULWDMSPlan50X, OLYMPUS) and a tube lens (MPlanAPO20X, MITUTOYO) to obtain a 4-f imaging system as shown in Fig. 1(b). The AMVHGs, which is recorded in photopolymer material phenanthrenequinone poly (methyl methacrylate) (PQ-PMMA), is located at the Fourier plane of the 4-f imaging system. In this study, AMVHGs consist of three angularly multiplexed gratings.

Grating vector of each gratings in AMVHGs, ${\overset{⇀}{K}}_{gi}$, can be calculated as^14^^,^^18^

$${\overset{⇀}{K}}_{gi}={\overset{⇀}{k}}_{ri}-{\overset{⇀}{k}}_{si},\;i=1,2,3,$$ (13)

where ${\overset{⇀}{k}}_{ri}$ and ${\overset{⇀}{k}}_{si}$ represent the propagation vector of the reference and signal beam given by

$${\overset{⇀}{k}}_{ri}=k\;\sin\;\theta_{ri}\;\overset{⇀}{x}+k\;\cos\;\theta_{ri}\;\overset{⇀}{z},$$ (14)

$${\overset{⇀}{k}}_{si}=k\;\sin\;\theta_{si}\overset{⇀}{x}+k\;\cos\;\theta_{si}\overset{⇀}{z},$$ (15)

where $\theta_{ri}$ and $\theta_{si}$ are the incident angle of reference and signal beams, respectively, and k=2π/λ, where λ is the operation wavelength. Following the previous methods, the recording angles for the first grating are ($\theta_{r1}=30{}^{\circ}$ and $\theta_{s1}=30{}^{\circ}$).^14^ Keeping the identical signal beam angle as the first grating, reference beam angle for recording second and third gratings are $\theta_{r2}=32{}^{\circ}$ and $\theta_{r3}=34{}^{\circ}$, respectively.

The recording setup for AMVHGs and wavelength-coded volume holographic gratings (WC-VHGs) was built up following our previous work.^19^^,^^20^ The setup uses the beam splitter to divide the argon ion (Ar+) laser light source into the reference and signal beams. In the signal beam, the spherical wavefront is generated by two microscope objectives. The axial position of the first objective lens (0.65 NA) is controlled by a miniature motorized linear stage. Therefore, the linear stage adjusts the position of the objective lens with axial displacement (Δz=50  μm) of objective lens for generating different wavefront of signal beam for each multiplexed grating.^14^

In imaging system, a AMVHG, under Bragg-match condition,^3^^,^^18^^,^^21^ acts like a multifocus lens and simultaneously display laterally separated multidepth images of volumetric samples onto the CCD as schematically shown in Fig. 1. In DPC-VHM system, when object is asymmetrically illuminated by projecting half-circle patterns on TFT-panel, with the help of AMVHGs, three asymmetrically illuminated images of object corresponding to three different axial locations are simultaneously acquired in a single shot. By changing half-circle pattern to opposite direction, the other set of pair-wise images from different focal planes is captured. Then, based on DPC image operation given by Eqs. (1315), multiplane DPC images can be obtained using the acquired pair of the asymmetric illumination images from different planes, without axial scanning.

### 2.3 WC-VHGs-Based DPC-VHM

**Fig. 2:**
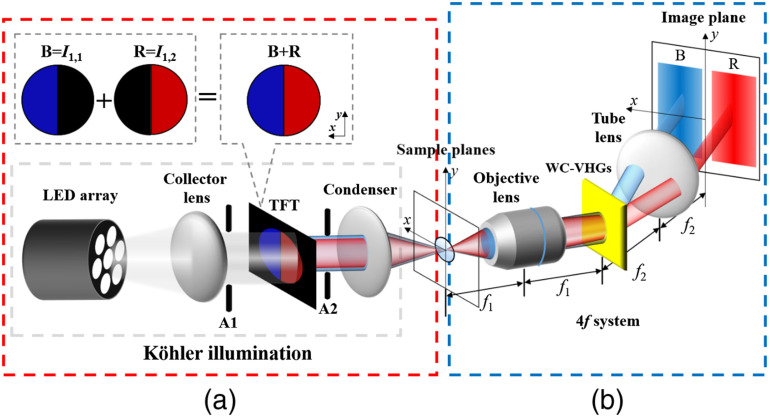
Schematic diagram of the DPC-VHM using WC-VHGs. (a) Asymmetric illumination within the highlighted red dashed box and (b) WC-VHGs imaging within the highlighted blue dashed box. The TFT-panel is used to produce combined blue–red half-circle pattern. White LED source to obtain color illumination (WC-VHGs, wavelength-coded volume holographic gratings).

To further reduce the image acquisition time, a second configuration based on WC-VHGs is presented that helps in simultaneously acquiring two different direction asymmetric illumination images with two different colors in single shot.^20^ At present, only a single-depth WC-VHGs is shown in this work. In Fig. 2, a white LED light source (LIUCWHA, THORLAB, λ=425 to 700 nm) for illumination is used. To generate light with different color illuminations from the TFT-panel, a red–blue half-circle mask is projected onto the TFT-panel, which generates two light patterns on object corresponding to two different wavelengths. The two-color amplitude mask is equivalent to two half-circles ($I_{1,1}$) and ($I_{1,2}$), respectively. For our case, the diameter of the circular pattern is around 3 mm. WC-VHG is located in the Fourier plane of the 4-f imaging system that includes two multiplexed gratings designed for specific wavelength λ1=633  nm and λ2=488  nm, respectively. The WC-VHG for single depth is designed and recorded in PQ:PMMA. According to Eqs. (57), the recording angles for first grating are ($\theta_{r1}=45{}^{\circ}$ and $\theta_{s1}=45{}^{\circ}$) and for the second grating are ($\theta_{r2}=45{}^{\circ}$ and $\theta_{s2}=66.524{}^{\circ}$). In addition, the configuration is designed for one depth DPC image, so the WC-VHGs recording axial displacement (Δz) is zero during multiplexing.

Under Bragg-match condition, WC-VHGs display different wavelength images with different diffracted angles to the corresponding laterally separated position onto the CCD. Therefore, the DPC-VHM using WC-VHGs simultaneously acquires two different direction asymmetric illumination images with two different colors in single shot.

## 3 Results

### 3.1 AMVHGs-Based DPC-VHM

**Fig. 3:**
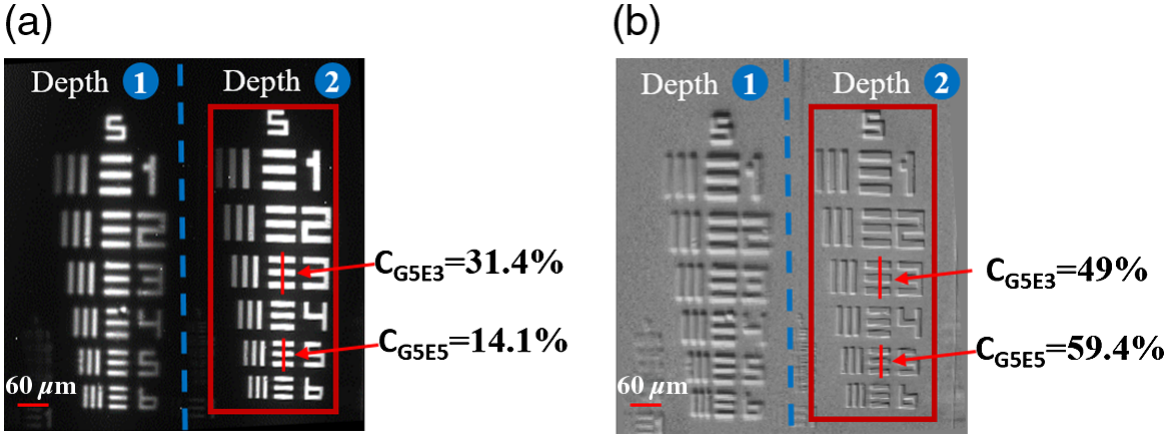
Comparison of multidepth images of AFRC. (a) Bright-field volume holographic microscope images of resolution chart. (b) Corresponding DPC-VHM images of resolution chart. Red box indicates the contrast ratio of different elements.

Figure 3(a) shows experimentally obtained multiplane images of the Air Force Resolution Chart (AFRC), under bright-field condition, which can easily be acquired by illuminating TFT-panel with a full-circle symmetric pattern. In Fig. 3(a), two multiplane images are separated by 50  μm in axial direction. The separation depth of the imaging plane obtained from AMVHGs can be controlled by signal beam parameters during the recording process. Since the AFRC is a thin sample, the image at depth 2 is in focus while the image at depth 1 is out-of-focus. The lateral features up to 2.46  μm can be well resolved in our experiment. Figure 3(b) shows the corresponding multiplane DPC images obtained by the proposed DPC-VHM system, using the asymmetric half-circle illumination patterns. Compared with the bright-field volume holographic microscope images shown in Fig. 3(a), the contrast ratio of high-frequency features with DPC-VHM [Fig. 3(b)] is obviously improved.

**Fig. 4:**
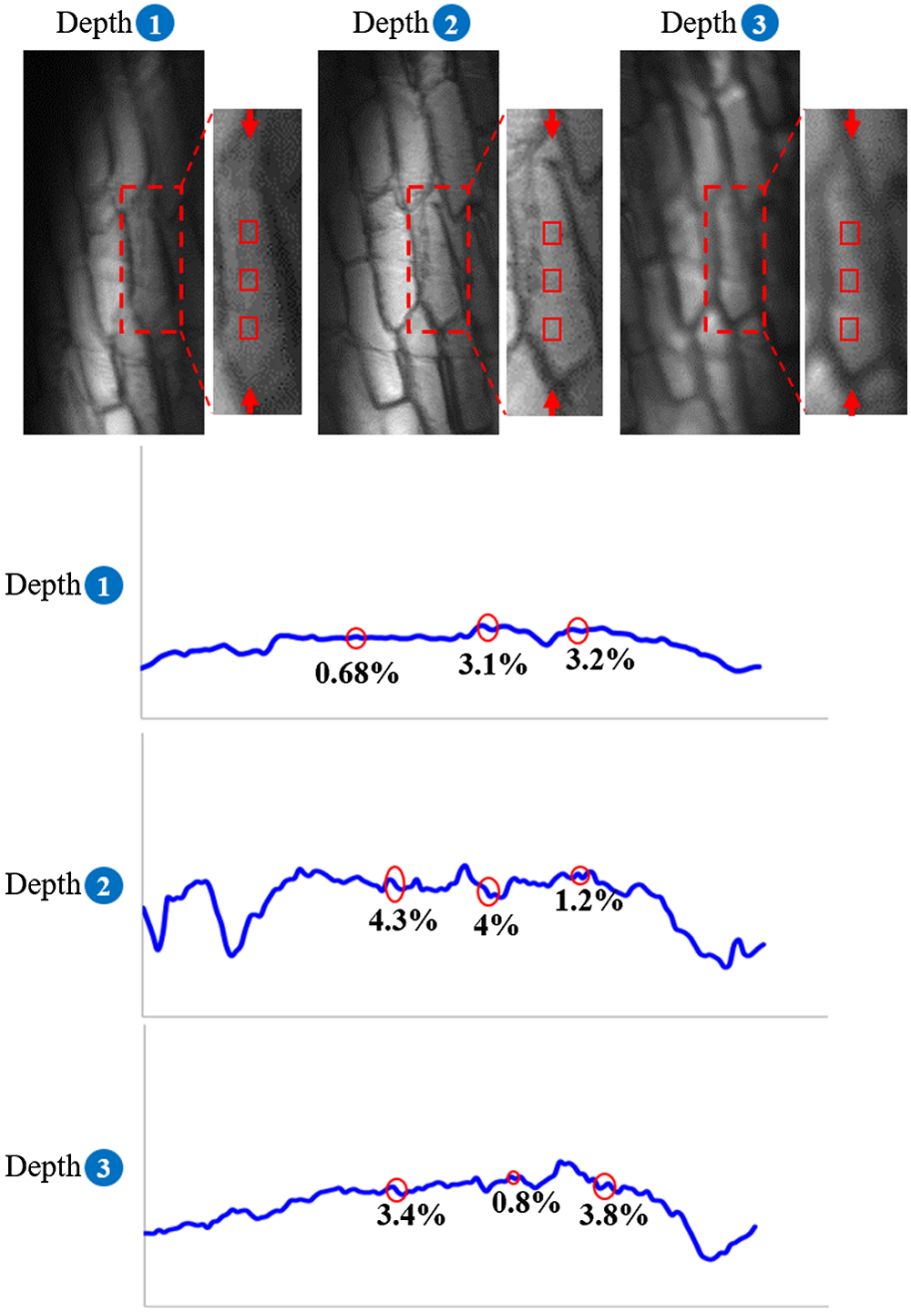
Three depth-resolved images of onion skin obtained simultaneously by the bright-field volume holographic microscope. The bottom panel corresponds to the contrast ratio of red box regions at the top panel in each depth.

**Fig. 5:**
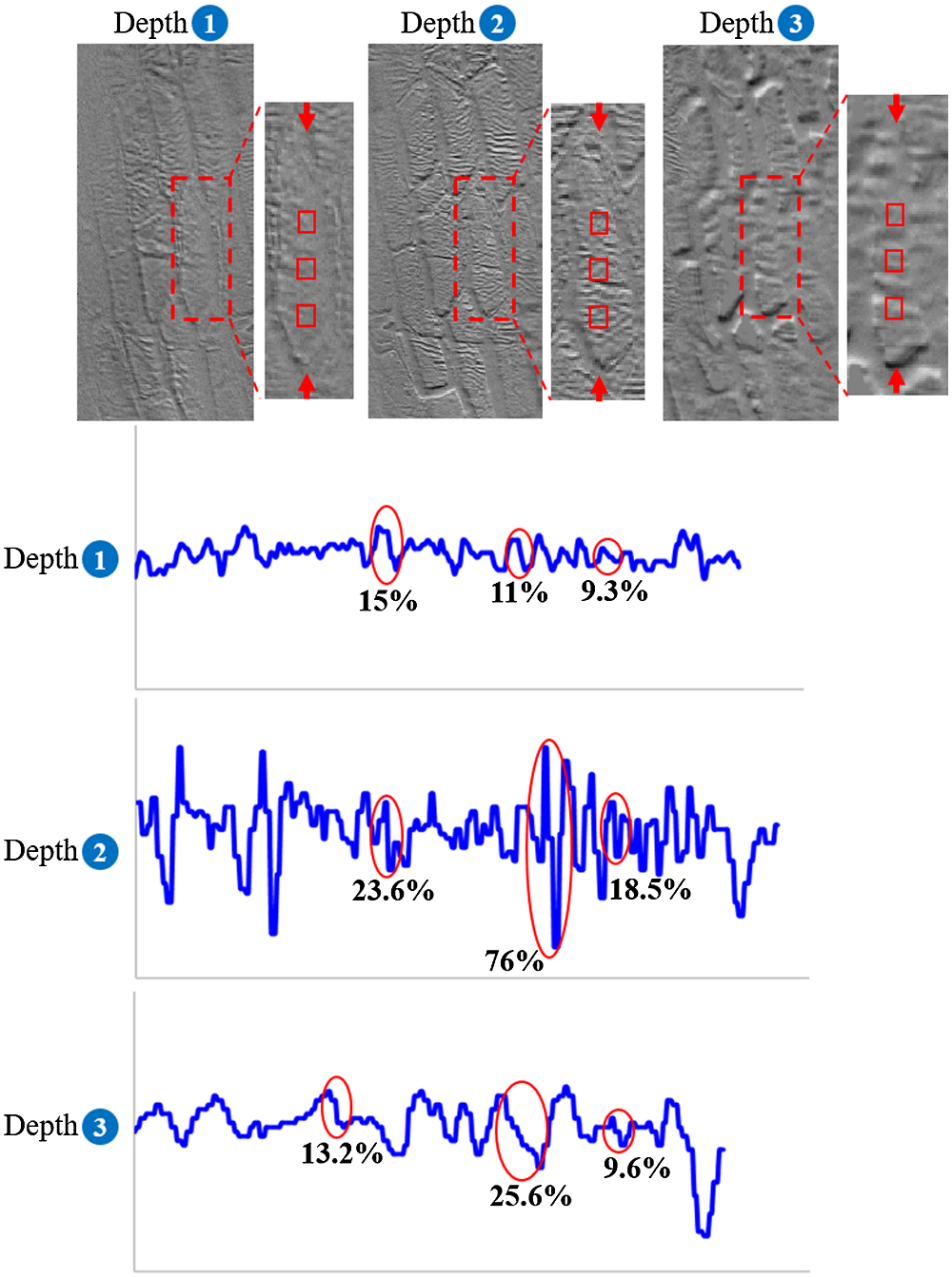
Three depth-resolved images of onion skin obtained simultaneously by the proposed DPC-VHM. The bottom panel corresponds to the contrast ratio of red box regions at the top panel in each depth.

In addition, we use onion skin samples to test the ability of the DPC-VHM system to resolve weak phase features. Compared to Fig. 4 using a bright-field volume holographic microscope, Fig. 5 displays the DPC-VHM images, where fine features within an onion skin sample are significantly enhanced at three different depths. Figures 4 and 5 display the contrast ratio of the three selected regions of interest corresponding to the same locations between the bright-field volume holographic microscope and DPC-VHM, where the red box represents the selected features along the vertical line for three depths. Separation between each depth is 50  μm. At depth 2, at selected regions contrast is improved from 4.3%, 4%, 1.2% to 23.6%, 76%, 18.5%, respectively, by DPC-VHM.

**Fig. 6:**
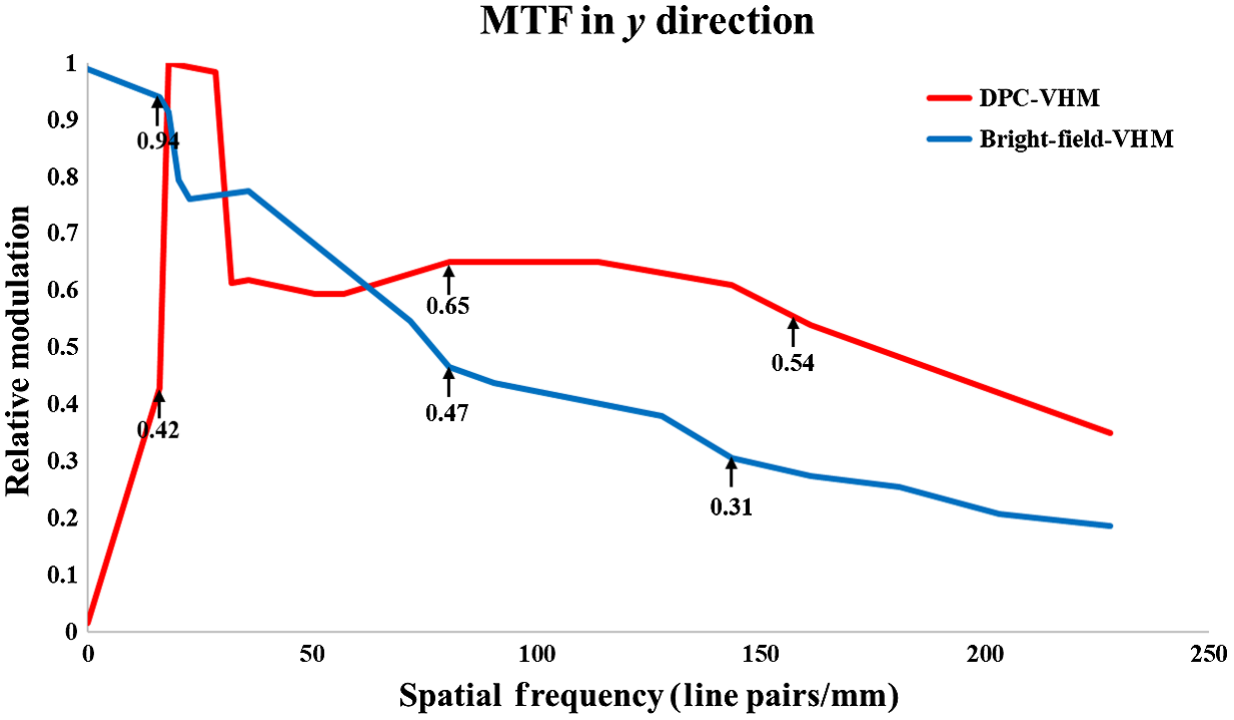
Relative MTF measurement in y direction (tangential direction) between the bright-field volume holographic microscope and DPC-VHM. The measurement result shows that the DPC-VHM not only can suppress the DC component (low-frequency part) but also can obviously enhance the high frequency information.

To precisely compare the resolution and performance of our systems, modulation transfer function (MTF) analysis was performed. The 1951 AFRC was introduced as an object. A comparison of MTF for the proposed DPC-VHM and bright-field volume holographic microscope is shown in Fig. 6. The MTF curve for the DPC-VHM clearly demonstrates the low spatial frequency term component is reduced, and contrast is obviously enhanced at the high-frequency region.

### 3.2 WC-VHGs Based DPC-VHM

**Fig. 7:**
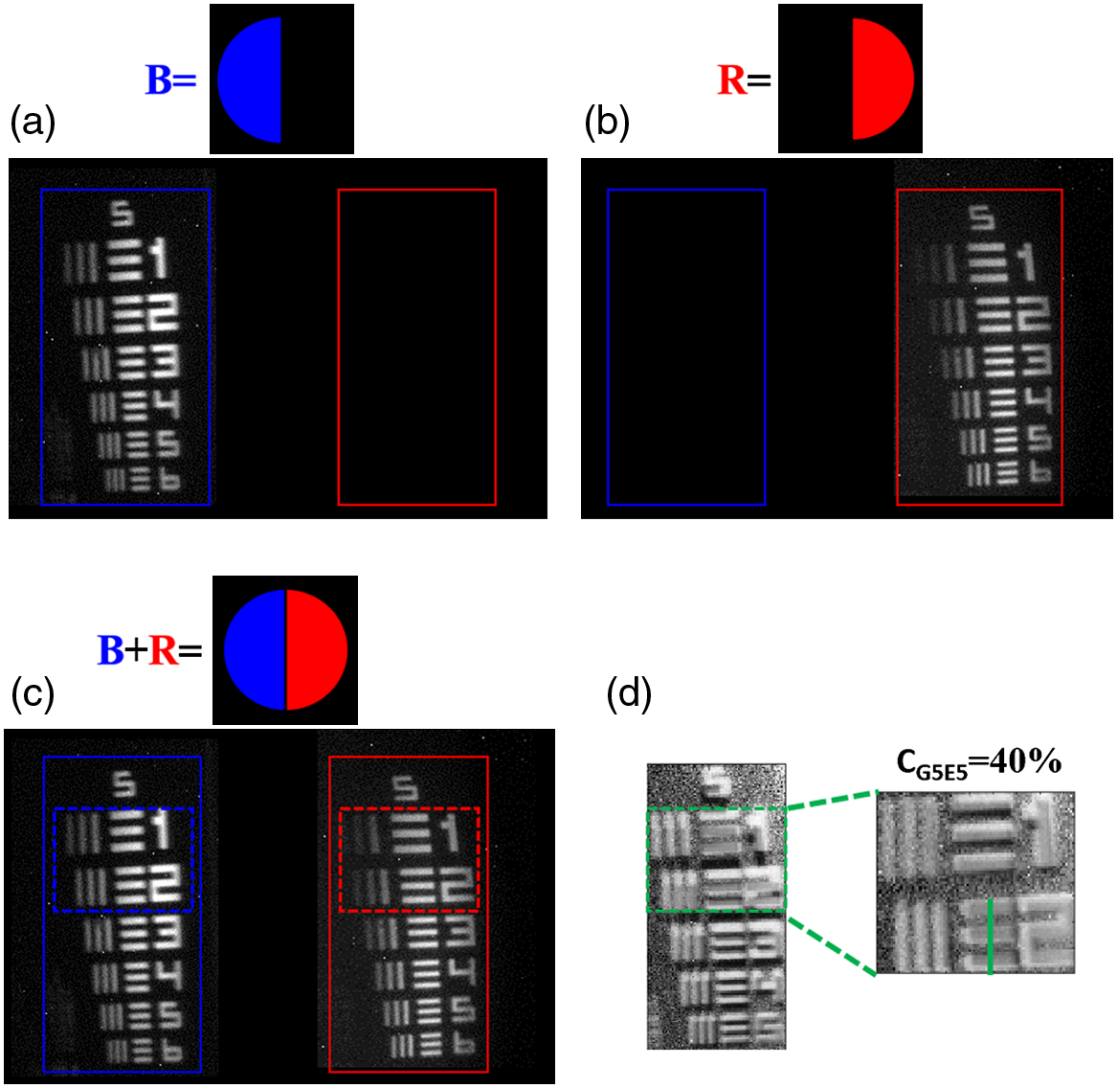
Image results of the AFRC obtained by the DPC-VHM using WC-VHGs. (a) The image obtained by projecting the blue half-circle mask onto the TFT-panel. (b) The image obtained by projecting the red half-circle (in the opposite direction) mask onto the TFT-panel. (c) The asymmetric illumination image acquired from the blue and red combined circle on the same TFT-panel. (d) DPC images of resolution target and zoomed-in image from the dash green box.

In Fig. 7, the wavelength selective property of the WC-VHGs has been verified. The TFT-panel is illuminated with white LEDs and it acts as a color filter. A half-circle mask with blue and red color is projected on to TFT-panel sequentially and images of objects are obtained. According to the diffractive properties of WC-VHGs (which consists of two gratings designed for the red and blue color), the blue and red images can diffract into specific lateral position onto the CCD plane as shown in Figs. 7(a) and 7(b). Figure 7(c) demonstrates the image of a resolution chart with the blue and red combined circle project on to TFT-panel. Figure 7(d) represents the DPC image of AFRC at a specific depth, and dash green box shows the zoomed-in DPC image. At present, WC-VHGs have been demonstrated to obtain only single depth image, which can be further increased by recording multiplexed WC-VHGs.

The x-axis field of view of DPC-VHM depends on the bandwidth of the light source. For the first configuration, we used the broadband green LED light source (LIU525B, THORLAB) that has the bandwidth of about 80 nm. In the AMVHGs-based DPC-VHM, the field of view for each depth in x direction is around 350  μm. For the second configuration, we choose the white LED light source (LIUCWHA, THORLAB) that has the emission spectrum range 425 to 700 nm. In the WC-VHGs-based DPC-VHM, the field of view for each color image in x direction is 1200  μm. Volume holographic grating has degeneracy in the y direction. The field of view in the y direction is decided by the size of the limiting aperture. The lateral and axial resolutions of our system depend on the numerical aperture (NA) of objective and the wavelength of illumination light. In the two DPC-VHM configurations, we use the same objective lens (ULWDMSPlan50X, OLYMPUS) with NA=0.55. In the experimental results, the lateral features up to 2.46  μm can be well resolved by our DPC-VHM. The depth separation for two planes for our DPC-VHM is about 50μm that can be controlled by the recording parameters of volume holograms. We followed the previous work for the preparation of the volume holographic substrate and the recording of hologram.^14^^,^^19^ The axial displacement (Δz) for the signal beam is 50  μm during two multiplex gratings. By increasing the multiplex gratings inside the volume hologram, the measurable range in depth of DPC-VHM can be increased. For example, in the previous work, we show a depth range around 400  μm for large capacity multiplexed gratings.^14^

## 4 Conclusion

Two new DPC-VHM configurations are experimentally demonstrated. Two different kinds of volume holographic gratings are adopted for this purpose. AMVHGs, in DPC microscopy, help in acquiring multidepth-resolved non-axial scanning DPC images. MTF analysis of system shows that the low spatial frequency term component is reduced, whereas the contrast is enhanced for most of the frequencies. Image contrast can be significantly enhanced using our DPC-VHM as compared to the bright-field volume holographic microscope. We found that the DPC-VHM can simultaneously enhance contrast of fine features within a volumetric sample, such as onion skin, at three depths. In addition, we demonstrate that single-shot DPC microscopy is possible with the help of WC-VHGs. Our illumination system is dynamic and multipurpose, and offers many advantages. It can work in different microscopic modalities under different illumination conditions. A bright-field mode, phase contrast mode, and DPC mode can be adapted by just manipulating the mask on the TFT-panel. The presented configurations may be used for quantitative DPC measurements^2^ using different computational algorithms.
